# PReS-FINAL-2138: Abatacept (orencia) in treatment of different types of juvenile idiopathic arthritis

**DOI:** 10.1186/1546-0096-11-S2-P150

**Published:** 2013-12-05

**Authors:** ES Zholobova, OU Konopelko, LA Galstyan, OS Rozvadovskaya, MN Nikolaeva

**Affiliations:** 1 Department Of Pediatric Rheumatology, First Moscow Medical State University I.M. Sechenov, Moscow, Russian Federation

## Introduction

Abatacept (ABA) - a new generation biological DMARD. It was approved in Russia in 2009. ABA selectively modulates T-cell activation, which seems to play the key role in the pathogenesys of Juvenile Idiopathic Arthriris (JIA). Efficacy and safety of ABA was assessed in AWAIKEN trial in different types of patients who had polyarticular course of JIA at the enrollment.

## Objectives

This prospective observation was aimed to assess effectiveness and safety of ABA in patients with different types and clinical variants of active JIA in real clinical practice.

## Methods

30 children with JIA were treated with ABA. 23 (77%) pts had polyarticular course of JIA and 7 (23%) - systemic JIA. 11 children were diagnosed with acute uveitis (totally 14 affected eyes). 28 (93%) of pts were female. All patients had high degree (III) of disease activity. Mean disease duration was 6,1 ± 3,2 yr. 29 patients previously had an inadequate response to 2 or more non-biological, one child with heavy course of systemic JIA was treated with infliximab during 1 year before starting ABA. Treatment effectiveness was assessed according to the ACR Pediatric criteria: ACR pedi 30, ACR pedi 50, acrpedi 70, and ACR pedi 90 after 6 and 12 months of abatacept therapy. All patients received ABA in dose 10 mg/kg at day 1, week 2 and 4 and every 4 weeks thereafter.

## Results

In the group of pts with systemic JIA after 6 months of ABA treatment 6 of 7 patients (86%) achieved ACR Pedi 30 response, 5 (71%) - ACR Pedi 50, and 3 (43%) - ACR Pedi 70. 12 months data was available for 5 patients (others are still on treatment but less than 12 moths). Four of five pts (80%) achieved ACR pedi-30, 3(60%) - ACR Pedi 50 response, 2 (40%) ACR pedi 70, and 1 (20%) ACR pedi 90.

In the group of pts with poliarticular course of JIA treatment effectiveness was assessed in 22 pts. One girl had experienced an infusion-related reaction (potentially associated with hypersensitivity) after 3^rd ^infusion and ABA treatment was stopped. After 6 months 20 of 22 pts (91%) achieved ACR pedi 30 response, 17 (77%) - ACR pedi-50, 9 (41%) - ACR pedi-70, 2 (9%) - ACR pedi 90. 14 of 22 patients with polyarticular JIA have completed 12 months of ABA treatment. 12 of them (86%) achieved ACR pedi 30 response, 11 (78%) - ACR pedi 50, 8 (57%) - ACR pedi 70 and 2 (14%) - ACR pedi 90. Abatacept also showed efficacy in treatment of JIA associated uveitis. After 6 months of treatment the count of affected eyes was significantly reduced. In 72% of patients complete remission of uveitis was achieved.

Adverse events were observed in 2 patients (6,7%): 1 case of infusion related reaction, 1 pts experienced common Herpes simplex infection (ABA treatment was stopped after 12 months).

## Conclusion

Abatacept have shown its effectiveness in the majority of patients with long standing JIA (systemic and polyarticular course) with high disease activity, and inadequate response to previous treatment with dmards. Better results were observed in patients with poliarticular JIA.

## Disclosure of interest

None declared.

